# Schizophrenia-like psychosis and gitelman syndrome: a case report and literature review

**DOI:** 10.1186/s40064-016-2579-5

**Published:** 2016-06-24

**Authors:** Bing Pan, Lijun Mou, Huichun Li, Weibo Liu, Ying Hu

**Affiliations:** Department of Psychiatry, Second Affiliated Hospital, Zhejiang University School of Medicine, 88 Jiefang Road, Hangzhou, 310009 Zhejiang China; Department of Nephrology, Second Affiliated Hospital, Zhejiang University School of Medicine, 88 Jiefang Road, Hangzhou, 310009 Zhejiang China

**Keywords:** Gitelman syndrome, Hypokalemia, Hypomagnesemia, Schizophrenia-like psychosis

## Abstract

**Introduction:**

Gitelman syndrome(GS) is a rare inherited tubular disorder which is characterized by hypokalemia, metabolic alkalosis, hypomagnesemia, and hypocalciuria. Here, we report a case of schizophrenia-like psychosis concomitant with GS and related literatures are reviewed.

**Case description:**

An 18-year-old male patient with 1-week history of auditory hallucinations, sense of insecurity, delusions of reference and feelings of being followed and controlled by others unknown, insomnia was admitted to Psychiatry department in December, 2013. Hypokalemia and hypomagnesemia were noted. He was diagnosed as schizophrenia-like psychosis. Treatment with paliperidone at the dose of 6 mg/day and magnesium and potassium supplementations was commenced. However, electrolyte disturbances failed to improve following psychosis remission. Therefore, other underlying diseases resulting in electrolyte disturbances were suspected. Along with hypokalemia and hypomagnesemia, additional investigation showing metabolic alkalosis, hypocalciuria, renal loss of potassium, were consistent with GS. Gene analysis revealed this patient carried out c. 2687 G > A homozygous mutation of exon 23 in the *SLC12A3* gene which led to p.Arg896Gln. Eventually, GS was identified. Thus, additional spironolactone (40 mg/day) combined with increased doses of oral potassium chloride sustained-release tablets (3.0 g/day) and potassium magnesium aspartate (0.3 g/day) were administered. During next half a year, fatigue resolved, paliperidone gradually tapered and eventually discontinued while psychosis maintained complete remission. His serum potassium was near normal (3.2–3.5 mmol/L), hypomagnesemia significantly improved (0.57–0.67 mmol/L).

**Discussion and evaluation:**

Electrolyte abnormalities secondary to GS might cause or contribute to development of neuropsychiatric symptoms. In turn, hypokalemia was common among acute psychiatric inpatients. As a consequence, when concomitant with psychosis, GS was readily concealed.

**Conclusion:**

Electrolyte disturbances are common in acute psychiatric patients. However, when electrolyte disturbances are not improved following psychosis remission, other underlying diseases such as GS should be considered.

## Background

Gitelman syndrome(GS) is a rare autosomal recessive tubular disorder first described by Gitelman et al. (1966). It is caused by mutation of *SLC12A3* gene, which encodes the Sodium/Chloride co-transporter (NCCT) in the distal convoluted tubule (Simon et al. 1996). GS is characterized by hypokalemia, metabolic alkalosis, hypomagnesemia and hypocalciuria. The majority of patients with GS do not present with symptoms until late childhood or adulthood (Naesens et al. 2004). Electrolyte abnormalities secondary to GS may cause or contribute to development of neuropsychiatric symptoms. In turn, hypokalemia is common among acute psychiatric patients (Lam et al. 2009). As a consequence, when concomitant with psychosis, GS is readily concealed. Here we report a case of schizophrenia-like psychosis in a 18-year-old boy with coexisting GS.

## Case description

An 18-year-old boy with 1-week history of auditory hallucinations, sense of insecurity, delusions of reference and feelings of being followed and controlled by others unknown, insomnia was admitted to Psychiatry department in December, 2013. He often heard many boys and girls commenting on him and criticising him sometimes. He suspected people were always discussing him and doing harm to him. These symptoms mentioned above made him sometimes just stare blankly, feel nervous, restless, tired, irritable, afraid of going outside. Insomnia subsequently developed. He was previously healthy and had no remarkable family history. He was enrolled in a high school. He did not smoke, drink alcohol or use illicit drugs. After admission to the hospital, physical examination was normal. On mental state examination, the patient was oriented and cooperative. Positive and Negative Symptoms Scale (PANSS) scored 95 which indicated he was in acute psychosis. His full scale of Weschler Adult Intelligence was 98 which indicated his intelligence was normal. His electrocardiogram, brain magnetic resonance imaging were normal. The laboratory investigations showed hypokalemia (3.17 mmol/L), hypomagnesemia (0.48 mmol/L).

According to his symptoms and investigations, the patient was diagnosed with schizophrenia-like psychosis and was treated with paliperidone at the dose of 6 mg/day and magnesium and potassium supplementations. Two weeks later, anxiety ameliorated, quality of sleep improved, hallucination and delusion resolved. The PANSS scores decreased to 41.His serum potassium increased to 3.23 mmol/L, magnesium level was still at 0.48 mmol/L. His electrolyte disturbances were thought to be caused by acute psychosis and were expected to recover soon, the patient was discharged with oral potassium and magnesium supplementations.

During the regular following up, the patient felt a little fatigue as before while his psychosis maintained complete remission. Two weeks later after discharge, laboratory studies showed worsened hypokalemia (2.99 mmol/L) and hypomagnesemia (0.49 mmol/L) despite potassium and magnesium supplementations. He had no history of diuretic, laxative and licorice intake, no episode of anorexia, vomiting and diarrhea. Considering that his psychosis and other common conditions failed to explain his electrolyte disturbances, we consulted a nephrologist who suspected this patient might had GS, further examinations were subsequently ordered. The results of hypokalemia, hypomagnesemia, metabolic alkalosis, hypocalciuria were consistent with GS (Table 1).

**Table 1:** Results of the laboratory investigations

Variable		Reference range
*Serum biochemistry*		
Sodium (mmol/L)	138.4	135 to 145
Potassium (mmol/L)	2.99	3.5 to 5.5
Urea (mmol/L)	4.10	2.80 to 7.2
Creatinine (μmol/L)	59	53 to 133
Fasting blood glucose (mmol/L)	4.51	3.89 to 6.11
Calcium (mmol/L)	2.37	2.08 to 2.60
Phosphate (mmol/L)	1.31	0.81 to 1.45
Magnesium (mmol/L)	0.49	0.73 to 1.06
Albumin (g/L)	52	35.0 to 52.0
Uric acid (μmol/L)	436	208 to 428
Alanine aminotransferase (U/L)	20	<45
Aspartate aminotransferase (U/L)	21	<35
Intact parathyroid hormone(pg/ml)	26.86	15.00 to 65.00
h-TSH (mIU/L)	4.17	0.35 to 4.94
Growth hormone (ng/mL)	<0.05	<4.80
Aldosterone (standing, ng/L)	192.49	65.00 to 296.00
Plasma rennin activity (standing, μg/L/h)	0.29	0.93 to 6.56
*Arterial blood gas*		
PH value	7.46	7.35 to 7.45
PaCO2 (mmHg)	46.7	36 to 44
Actual bicarbonate (mmol/L)	28.7	22 to 26
Base excess (mmol/L)	3.6	−3.0 to +3.0
Standard bicarbonate (mmol/L)	27.6	22 to 26
*Urinalysis*		
PH value	7.49	4.6 to 8.0
Sodium (mmol/24 h)	238	65.00 to 296.00
Potassium (mmol/24 h)	114.9	25 to 100
Calcium to creatinine ratio (mmol/mmol)	0.058	>0.2

**Fig. 1:**
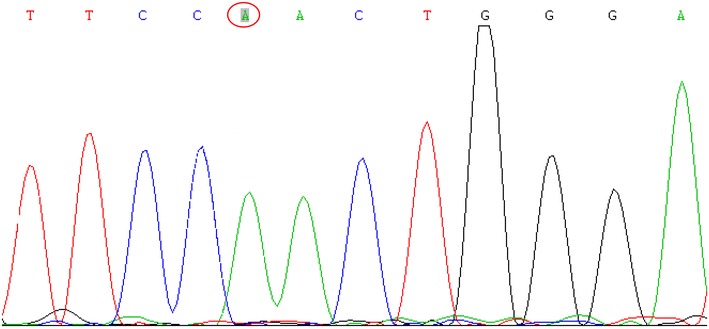
The mutated sequence of NCCT polymerase chain reaction (PCR) fragment. The *red circle* indicates a homozygous c.2687 G > A mutation of exon 23 in *SLC12A3* which led to p.Arg896Gln

In order to verify this diagnosis, gene analysis was performed. Genomic DNA was isolated and purified from peripheral blood of the subject and used for polymerase chain reaction (PCR) amplification of individual exons of the *SLC12A3* gene. Twenty-six pairs of oligonucleotide primers were generated to amplify all 26 exons and flanking intronic regions. PCRs were performed in a 50 μL volume containing 1 μL genomic DNA, 2 μL primers, 1 μL deoxyribonucleotide triphosphate(dNTP), and 5 μL 10 × 9 Taq buffer, 5 μL MgCl2, 2.5 U Taq enzyme, and 35.5 μL ddH2O. PCR was performed using standard conditions with an initial denaturation step at 95 °C for 3 min, subsequently followed by 35 cycles with denaturation at 94 °C for 30 s, annealing at 55–60 °C for 35 s and elongation at 72 °C for 40–50 s. Sanger direct sequencing was performed on an ABI 3730XL automated DNA sequencer (Applied Biosystems Division, Life Technologies, CA, USA) by Sangon Biotech Shanghai Co., (Shanghai, China). GenBank Accession Number NC_000016, was used as reference sequences. The numbering of a nucleotide starts at the first adenine of the translation initiation codon. This patient carried out c. 2687 G > A homozygous mutation of exon 23 in the *SLC12A3* gene which led to p.Arg896Gln which was already known as pathogenic mutation in the previous report (Kurschat et al. 2003) (Fig. 1).

Eventually, the diagnosis of GS was established. Thus, additional spironolactone (40 mg/day) combined with increased doses of oral potassium chloride sustained-release tablets (3.0 g/day) and potassium magnesium aspartate (0.3 g/day) were administered. During next half a year, fatigue resolved, paliperidone gradually tapered and eventually discontinued while psychosis maintained complete remission. His serum potassium was near normal (3.2–3.5 mmol/L), hypomagnesemia significantly improved (0.57–0.67 mmol/L).

## Discussion and evaluation

This patient with classical psychotic symptoms was diagnosed as schizophrenia-like psychosis at presentation. Hypokalemia and hypomagnesemia were noted. However, electrolyte disturbances failed to improve following psychosis remission which prompted us to suspect the electrolyte disturbances were caused by other underlying diseases. Eventually, GS was identified by gene analysis. To our knowledge, this is the first report of schizophrenia-like psychosis concomitant with GS.

GS is a rare disease which is initially considered as a mild type of the salt-losing tubular disorder (Sampathkumar et al. 2010). A later study demonstrated that GS is not an asymptomatic disease. It adversely affects quality of life in patients with GS principally resulted from secondary electrolyte abnormalities, to at least the same degree as more significant diseases like hypertension, diabetes, and cardiac conditions (Cruz et al. 2001). Hypokalaemia alone was associated with the development of central pontine myelinolysis which produced psychiatric symptoms such as changes in the level of consciousness and behaviour or cognitive dysfunction (Bahr et al. 1990; Sugimoto et al. 2003). Hypomagnesemia also led to clinically significant signs and symptoms when serum levels fall below 0.5 mmol/L (1.2 mg/dL). Clinical manifestations of hypomagnesemia that promptly led to medical attention involve neuromuscular hyperexcitability that may range from tremors, fascicula-tion, tetany, to convulsions, and neuropsychiatric disturbances including apathy, delirium, and even coma (Pham et al. 2014). Therefore, psychosis and electrolyte abnormalities secondary to GS manifested overlapping symptoms which would conceal the coexistence of GS.

In turn, hypokalemia was common among acute psychiatric patients (27.7 %), both agitation and the use of antipsychotics were postulated to contribute to the high prevalence of hypokalemia among acutely ill psychiatric patients (Lam et al. 2009). Antipsychotics were found to block the potassium efflux channel and prohibit intracellular potassium from shifting into the extracellular compartment, they have been reported to cause hypokalemia when being taken in excess or overdose (Malik et al. 2005). Hypokalemia already developed before the usage of paliperidone and persisted after discontinuation, so hypokalemia was not caused by antipsychotics in this patient. Agitation was postulated to stimulate the surge of serum adrenaline, which via the over-stimulation of beta-adrenaline receptors, caused influx of potassium into cells (Lam et al. 1999). Under this condition, hypokalemia might recover spontaneously following agitation remission. At first, we considered his electrolyte disturbance was only caused by agitation. But the electrolyte disturbances did not resolve after his psychosis remission which prompted us to investigate other causes. Eventually, the diagnosis of GS was established. With intensive potassium and magnesium supplementation and additional spironolactone, electrolyte disturbances significantly improved.

## Conclusion

Conclusively, electrolyte disturbances are common in acute psychiatric patients. However, when electrolyte disturbances are not improved following psychosis remission, other underlying diseases such as GS should be considered.

## Abbreviations

GS: gitelman syndrome
NCCT: sodium/chloride co-transporter
PCR: polymerase chain reaction
PANSS: Positive and Negative Symptoms Scale

## References

[CR1] Bahr M, Sommer N, Petersen D, Wietholter H, Dichgans J (1990). Central pontine myelinolysis associated with low potassium levels in alcoholism. J Neurol.

[CR2] Cruz DN, Shaer AJ, Bia MJ, Lifton RP, Simon DB, Gs Yale (2001). Gitelman’s syndrome revisited: an evaluation of symptoms and health-related quality of life. Kidney Int.

[CR3] Gitelman HJ, Graham JB, Welt LG (1966). A new familial disorder characterized by hypokalemia and hypomagnesemia. Trans Assoc Am Physicians.

[CR4] Kurschat C, Heering P, Grabensee B (2003). Gitelman’s syndrome: an important differential diagnosis of hypokalemia. Dtsch Med Wochenschr.

[CR5] Lam RW, Carter D, Misri S, Kuan AJ, Yatham LN, Zis AP (1999). A controlled study of light therapy in women with late luteal phase dysphoric disorder. Psychiatry Res.

[CR6] Lam MH, Chau SW, Wing YK (2009). High prevalence of hypokalemia in acute psychiatric inpatients. Gen Hosp Psychiatry.

[CR7] Malik AR, Wolf PK, Ravasia S (2005). Hypokalemia from risperidone and quetiapine overdose. Can J Psychiatry.

[CR8] Naesens M, Steels P, Verberckmoes R, Vanrenterghem Y, Kuypers D (2004). Bartter’s and Gitelman’s Syndromes: from Gene to Clinic. Nephron Physiol.

[CR9] Pham PC, Pham PA, Pham SV, Pham PT, Pham PM, Pham PT (2014). Hypomagnesemia: a clinical perspective. Int J Nephrol Renovasc Dis.

[CR10] Sampathkumar K, Muralidharan U, Kannan A, Ramakrishnan M, Ajeshkumar R (2010). Childhood Bartter’s syndrome: an Indian case series. Indian J Nephrol.

[CR11] Simon DB, Nelson-Williams C, Bia MJ, Ellison D, Karet FE, Molina AM (1996). Gitelman’s variant of Bartter’s syndrome, inherited hypokalaemic alkalosis, is caused by mutations in the thiazide-sensitive Na-Cl cotransporter. Nat Genet.

[CR12] Sugimoto T, Murata T, Omori M, Wada Y (2003). Central pontine myelinolysis associated with hypokalaemia in anorexia nervosa. J Neurol Neurosurg Psychiatry.

